# P-1258. Estimation Vancomycin Area Under the Curve (AUC) Using a Bayesian Model Software for Peripartum Patients Receiving Group B Streptococcal Prophylaxis

**DOI:** 10.1093/ofid/ofaf695.1449

**Published:** 2026-01-11

**Authors:** Reshma George, Juby Roy, Thien-Ly Doan, Brian Chung, Barbara Kamel, Sumeet Jain, Patricia Saunders-Hao, Henry Donaghy

**Affiliations:** Donald and Barbara Zucker School of Medicine at Hofstra/Northwell, Dayton and Karen Brown Division of Infectious Diseases, New Hyde Park, New York; Donald and Barbara Zucker School of Medicine at Hofstra/Northwell, Dayton and Karen Brown Division of Infectious Diseases, New Hyde Park, New York; Long Island Jewish Medical Center, New Hyde Park, NY; Long Island Jewish Medical Center, New Hyde Park, NY; Long Island Jewish Medical Center, New Hyde Park, NY; North Shore University Hospital, Westbury, NY; North Shore University Hospital, Westbury, NY; Donald and Barbara Zucker School of Medicine at Hofstra/Northwell, Dayton and Karen Brown Division of Infectious Diseases, New Hyde Park, New York

## Abstract

**Background:**

To decrease the risk Group B *Streptococcus* (GBS) maternal colonization, The American College of Obstetricians and Gynecologists (ACOG) recommends the use of intrapartum IV vancomycin for in those with documented high risk penicillin allergies when the isolate is clindamycin resistant and where the risk for allergic reaction is unknown. The recommendation for vancomycin is 20 mg/kg IV every 8 hours (maximum of 2 grams/dose). Although the courses of vancomycin are typically short, there is still concern that high doses may be excessive, especially in this patient population with altered pharmacokinetics. The analysis aims to estimate the area under the curve (AUC) of vancomycin prescribed for intrapartum GBS prophylaxis.

**Methods:**

This is a quality improvement analysis that estimated the vancomycin AUC of intrapartum patients from 2021 to 2024. Patients were adult patients admitted to Long Island Jewish Medical Center or North Shore University Hospital. A medication list was generated using the electronic medical record of admitted to labor and delivery, receiving vancomycin 1500 mg/dose or greater for GBS prophylaxis. Patients had to have at least 1 vancomycin levels drawn. Data collected included patient weight, allergy history, vancomycin dose and frequency, duration of therapy, laboratory findings. Using a Bayesian Model (e.g., PrecisePK), the area under the curve was calculated. Descriptive statistical analysis was performed.

**Results:**

Of the 367 screened, 31 patients met inclusion criteria. The mean weight was 98.4 ± 21.8 kg. All reported a beta-lactam allergy however 93.5% could have received a penicillin or cefazolin instead. Most were prescribed 20 mg/kg IV q8h (48.4%) and 15 mg/kg IV q8h (35.5%). The mean vancomycin daily dose was 5 ± 0.8 grams per day. Mean days of therapy was 2.3 ± 0.8 days. The mean AUC was estimated to be 748 ± 244 (range 385 - 1464). A total of 64.5% were deemed to be supratherapeutic (AUC > 600) while 32.3% were therapeutic (AUC 400 - 600). On average, patients could have received 1.5 grams less per day for a therapeutic AUC. Acute kidney injury was seen in 6 patients (19.4%).

**Conclusion:**

Supratherapeutic vancomycin AUC were seen in these intrapartum population. Acute kidney injury could be an unintended consequence that can be seen. A larger study is warranted.

**Disclosures:**

All Authors: No reported disclosures

